# Modulation of Aptamer–Ligand-Binding by Complementary Oligonucleotides: A G-Quadruplex Anti-Ochratoxin A Aptamer Case Study

**DOI:** 10.3390/ijms23094876

**Published:** 2022-04-28

**Authors:** Alexey V. Samokhvalov, Irina V. Safenkova, Sergei A. Eremin, Artem N. Bonchuk, Oksana G. Maksimenko, Nikolai N. Sluchanko, Anatoly V. Zherdev, Boris B. Dzantiev

**Affiliations:** 1 A.N. Bach Institute of Biochemistry, Research Center of Biotechnology, Russian Academy of Sciences, Moscow 119071, Russia; 03alexeysamohvalov09@gmail.com (A.V.S.); saf-iri@yandex.ru (I.V.S.); eremin_sergei@hotmail.com (S.A.E.); nikolai.sluchanko@mail.ru (N.N.S.); zherdev@inbi.ras.ru (A.V.Z.); 2 Faculty of Chemistry, M.V. Lomonosov Moscow State University, Moscow 119991, Russia; 3 Center for Precision Genome Editing and Genetic Technologies for Biomedicine, Institute of Gene Biology, Russian Academy of Sciences, Moscow 119334, Russia; errinaceus@rambler.ru (A.N.B.); maksog@mail.ru (O.G.M.)

**Keywords:** aptamers, G-quadruplex, DNA–DNA interactions, isothermal microcalorimetry, fluorescence anisotropy, circular dichroism, ochratoxin A

## Abstract

Short oligonucleotides are widely used for the construction of aptamer-based sensors and logical bioelements to modulate aptamer–ligand binding. However, relationships between the parameters (length, location of the complementary region) of oligonucleotides and their influence on aptamer–ligand interactions remain unclear. Here, we addressed this task by comparing the effects of short complementary oligonucleotides (ssDNAs) on the structure and ligand-binding ability of an aptamer and identifying ssDNAs’ features that determine these effects. Within this, the interactions between the OTA-specific G-quadruplex aptamer 1.12.2 (5′-GATCGGGTGTGGGTGGCGTAAAGGGA GCATCGGACA-3′) and 21 single-stranded DNA (ssDNA) oligonucleotides complementary to different regions of the aptamer were studied. Two sets of aptamer–ssDNA dissociation constants were obtained in the absence and in the presence of OTA by isothermal calorimetry and fluorescence anisotropy, respectively. In both sets, the binding constants depend on the number of hydrogen bonds formed in the aptamer–ssDNA complex. The ssDNAs’ having more than 23 hydrogen bonds with the aptamer have a lower aptamer dissociation constant than for aptamer–OTA interactions. The ssDNAs’ having less than 18 hydrogen bonds did not affect the aptamer–OTA affinity. The location of ssDNA’s complementary site in the aptamer affeced the kinetics of the interaction and retention of OTA-binding in aptamer–ssDNA complexes. The location of the ssDNA site in the aptamer G-quadruplex led to its unfolding. In the presence of OTA, the unfolding process was longer and takes from 20 to 70 min. The refolding in the presence of OTA was possible and depends on the length and location of the ssDNA’s complementary site. The location of the ssDNA site in the tail region led to its rapid displacement and wasn’t affecting the G-qaudruplex’s integrity. It makes the tail region more perspective for the development of ssDNA-based tools using this aptamer.

## 1. Introduction

Aptamers are single-stranded oligonucleotide receptor molecules that are in demand for biosensor development due to their simple structure, low cost, efficient renaturation, and the possibility of chemical synthesis and modification [1,2,3]. Ligand recognition is enabled by the tertiary structure of aptamers, in which G-quadruplexes are widely presented [4]. In G-quadruplexes, the guanine-enriched region of an oligonucleotide chain is assembled into a tetrad of several planar guanines stabilized by hydrogen bonds. The overall G-quadruplex assembly is maintained by metal cations such as Na^+^, K^+^, Mg^2+^, Ca^2+^ [5,6]. The advantages of G-quadruplexes, which determine their wide application, include their high resistance to many nucleases, thermodynamic stability, and an ordered arrangement of charges, which in some cases contributes to high-affinity ligand recognition [4,7].

To modulate the aptamer–ligand binding, short oligonucleotides complementary to different regions of the aptamer are widely used [8,9,10,11]. However, comparisons of such oligonucleotides that differ in the length and/or location of their binding sites are very rare [12,13,14,15] and are limited by searching for the best oligonucleotides for analytical use without discussion of the ssDNA-driven structural changes and features of the interactions that influence the ligand-binding ability. Therefore, the task of the present study was to compare the effects of short complementary oligonucleotides (ssDNAs) on the structure and ligand-binding ability of an aptamer and to identify the binding parameters that determine these effects.

**Figure 1:**
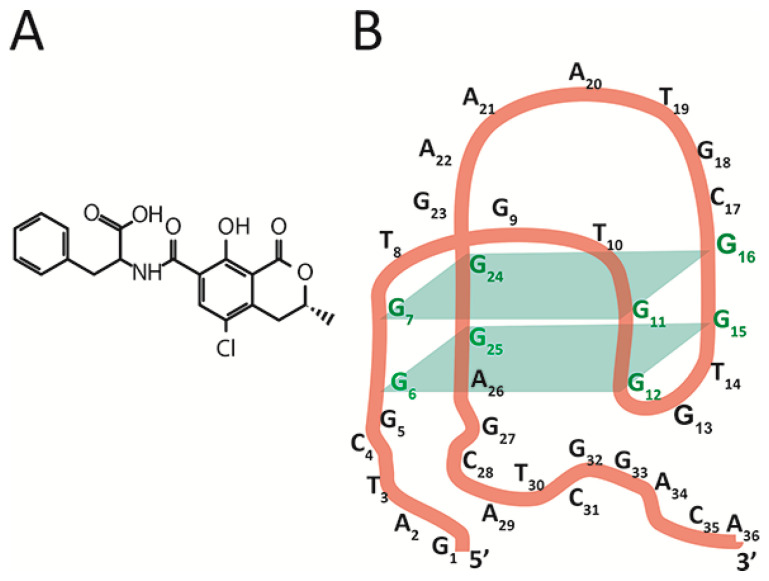
(**A**) Chemical structure of OTA. (**B**) The expected structure of the antiparallel G-quadruplex of OTA-binding aptamer 1.12.2.

The chosen object, aptamer 1.12.2, is a 36-nucleotide single-stranded DNA [16] capable of binding a low-molecular-weight toxicant, ochratoxin A (OTA) (see Figure 1B), a widespread contaminant of many agricultural plants that is characterized by its complex toxic effects on humans and animals [17]. For this aptamer, there are no direct data on the spatial structure, with only general principles of its structural organization being available [15,17,18,19,20]. The aptamer 1.12.2 is composed of an antiparallel G-quadruplex, a 5’-, and a 3’-tail. Fadock et al. suggested the structural organization of the aptamer [18] depicted in Figure 1B.

Antiparallel G-quadruplex formation and OTA binding are possible only in the presence of ions such as Mg^2+^ or Ca^2+^, and the contribution of structural elements of the aptamer beyond the G-quadruplex to OTA binding were also shown [16,19,20,21]. The advantages of the chosen model of aptamer are its reported high affinity with the ligand, the 1:1 stoichiometry of the resulting complex, and the possibility of comparison with earlier studies on different factors influencing the aptamer’s structure and ligand-binding properties [16,18,19,22].

In accordance with the objective, this study was carried out in the following order. To determine the relationship between the parameters of complementary ssDNAs and their influence on aptamer–ligand interactions, ssDNAs with different lengths and locations for their complementary regions were chosen. The interaction of each ssDNA with the aptamer was characterized using isothermal calorimetry (ITC). Fluorescence anisotropy (FA) was measured in kinetic and steady-state modes for fluorescein-labeled OTA in the presence of ssDNAs and aptamers. The dissociation constants of aptamer–ssDNA interactions were calculated using ITC and FA data and compared with ssDNA parameters. The influence of the ssDNA parameters on the aptamer’s structure and its OTA-binding properties was evaluated using circular dichroism (CD) spectroscopy.

This article considers the interactions of twenty one ssDNAs (5–10 bases) fully complementary to different regions of the aptamer, and the inhibition of the aptamer–ligand binding in the presence of these ssDNAs. The influence of the ssDNA parameters (the length and location of the complementary region) on the spatial structure and ligand-binding properties of the aptamer was inferred from the binding data.

## 2. Results and Discussion

### 2.1. Selection of Oligonucleotides Fully Complementary to the G-Quadruplex Aptamer

For the correct evaluation of the influence of ssDNA parameters on the aptamer–ligand binding ability and its structure, interfering interactions should be minimized. Namely, the ssDNA used should not form hairpins or dimers and should have only one complementary site. Compliance of the candidate ssDNA sequences with these requirements was checked. The Multiple Primer Analyzer was used to predict the number of binding sites and to evaluate their complementarity. Then, the absence of self-dimers and hairpins was checked by the OligoCalc; the minimum number of base pairs causing dimerization and formation of hairpins was taken as equal to three.

The range of the ssDNAs’ length was chosen to be from 5 to 10 nucleotides. Complementary regions of the same length have different interaction constants depending on the number of AT and GC pairs. Therefore, the chosen ssDNAs were characterized not only by their length and location but also by the number of hydrogen bonds formed with the aptamer in the complementary region.

Published data on single-nucleotide deletions/substitutions [16,19] and our studies on shortened aptamer variants (Table S1) demonstrate the contribution of all structural elements to the OTA-binding properties of the aptamer 1.12.2. Hence, both the G-quadruplex and the two tails were studied as ssDNAs’ binding targets. The chosen complementary sites for ssDNA binding were localized in one of the elements or were partially covering two adjacent ones.

As a result, the panel of 21 ssDNAs listed in Table 1 was selected. Hereinafter, ssDNAs are named by their position corresponding to the complementary region of the aptamer.

### 2.2. Comparison of Dissociation Constants of ssDNAs and OTA with Aptamer by Isothermal Microcalorimetry

Before characterization of the ssDNAs’ influence on aptamer–ligand binding, the aptamer–ssDNA interactions should be compared for the cases of OTA absence and presence. The ITC method was used for that purpose. In more detail, to register the heat caused by the interaction, aliquots of 21 ssDNAs and OTA were successively added to the aptamer. Heat generation was registered and thermodynamic parameters of the interaction were calculated.

**Figure 2:**
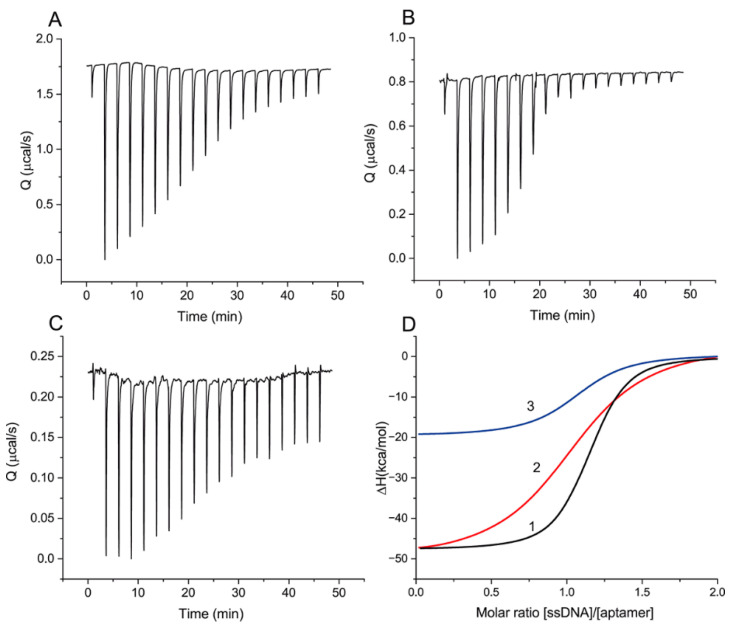
ITC measurements for sequential additions of ssDNA to aptamer: (**A**)—300 µM of ssDNA {1–8} to 25 µM of aptamer, (**B**)—100 µM of ssDNA {28–36} to 8 µM of aptamer, (**C**)—60 µM of OTA to 5 µM of aptamer. (**D**) The integrated heat plots for ssDNAs {1–8} (1) and {28–36} (2) and for OTA (3).

Figure 2 presents raw data of the heat resulting from each injection and the integrated heat plots for ssDNAs {1–8} and {28–36} and OTA, with different changes in heat values interpreted as differences in binding constants. A complete set of data for all ssDNAs is given in Supporting Information S2.

The obtained thermodynamic parameters are summarized in Table 2. The heat resulting from the addition of ssDNA {1–5} was too weak for reliable determination of the thermodynamic parameters of binding [23] and indicates a significantly lower K_D_ value than for the rest of the ssDNAs.

Table 2 and Figure 2 shows that all interactions of the aptamer with the ssDNAs and OTA are exothermic (∆H < 0) and correspond to the interaction stoichiometry (N) of 1 to 1. For the formation of DNA–DNA complexes, a comparable contribution of ∆H and T∆S to ∆G is typical [24], which agrees with our experimental data about the formation of aptamer–ssDNA complexes. ∆H contributes from 53.8% to 56.1% to the total ∆G in the studied ssDNA panel. The interaction of OTA with the aptamer has thermodynamic parameters different from the aptamer–ssDNA interaction. ∆H makes two-thirds of the total contribution to ∆G. At the same time, the reaction of the aptamer with OTA is associated with a lower heat release in comparison with all ssDNAs (except {1–5}), including those with an order of magnitude lower K_D_ than that for OTA ({1–8}, {12–18}, {19–27}, and {30–36}). The obtained results demonstrated the decrease in the ssDNA–aptamer dissociation constant with an increasing number of H-bonds formed between the aptamer and ssDNA in duplex.

### 2.3. Fluorescence Anisotropy of OTA-Flu Probe Reveals Diversity of ssDNA Inhibition Kinetics

**Figure 3:**
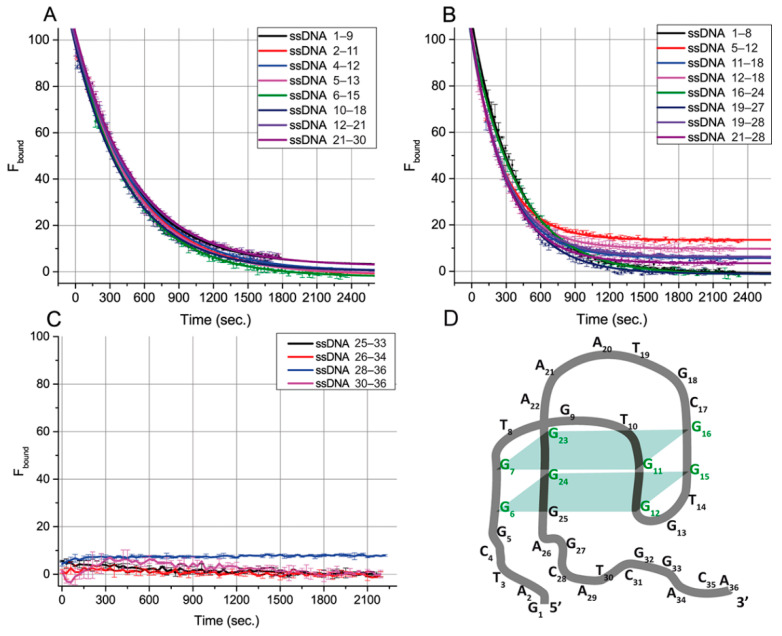
Dependences of the percentage of bound OTA-Flu on time in the presence of the excess of ssDNA overlapping with the G-quadruplex (**A**,**B**), with 3’-tail (**C**), or of the aptamer. (**D**) Structural model of the aptamer 1.12.2 consistent with FA results. The structure was drawn using the QGRS mapper software [25].

To characterize the influence of ssDNAs on aptamer–ligand-binding abilities, we used a fluorescein-labeled OTA derivative (OTA-Flu) and measured its fluorescence anisotropy. The kinetics of the transition between the aptamer complexes with OTA and ssDNA were evaluated to make additional conclusions about the features of the aptamer–ssDNA binding. The system containing the aptamer, OTA-Flu, and ssDNA was studied for this purpose using the FA technique. The measured FA values were transformed into the percentage of bound OTA-Flu (F_bound_). These experiments were implemented for 20 ssDNAs (Figure 3), except for ssDNAs only related to group I, {1–5}, for which no effect on FA was observed. The obtained dependences were approximated by the exponential decay function. The time needed for 50% decay of the aptamer–OTA-Flu complex (half-life), the decay rate, and the equilibrium time (i.e., the time during which a 99% change in FA occurs) were determined for all ssDNAs—see Table 3.

Based on Figure 3 and Table 3, all ssDNA can be divided according to the time required for complete inhibition of the aptamer–OTA-Flu binding. For sixteen ssDNAs from groups II, III, and IV, the aptamer–OTA-Flu complex was formed before the aptamer–ssDNA complex. Then, OTA-Flu was slowly (20–40 min) released—see Figure 3A,B. For four ssDNAs from groups IV and V, the formation of the aptamer–OTA-Flu complex was not detected—see Figure 3C. The aptamer–OTA-Flu interaction is inhibited by ssDNAs faster than we can detect it (the time delay before measurements was one minute). Similar rapid processes for OTA and OTA-Flu interacting with the aptamer were described in [26].

The correlation between the G-quadruplex’s overlap with ssDNA and the time for reaching the reaction equilibrium was studied. Different ssDNA:(OTA-Flu) ratios were tested to reach the complete inhibition of OTA-Flu binding, and for most cases, it was obtained (see Figure 3A,B). The comparison of eight ssDNAs (see Figure 3A) providing complete displacement of OTA-Flu under the same concentration of ssDNA (4 µM) showed that their equilibrium time was in the range from 36.0 to 39.7 min, regardless of the position of the G-quadruplex’s overlap or its length, see Table 3. 

The assignment of ssDNA {25–33} from group IV into group V needs additional consideration. This ssDNA as well as all ssDNA from group V ({26–34}, {28–36}, and {30–36}) rapidly reach equilibrium in their interaction with the aptamer–OTA complex—see Figure 3C. However, binding sites for group V are completely located at the aptamer’s 3’-tail. This is the reason to consider the 25th guanine (involved in the complementary interaction with the ssDNA {25–33}) as a part of the 3’-tail and not of the G-quadruplex, which contradicts the earlier published structure [18]—see Figure 1B. This consideration has been summarized by our proposition on the corrected structure of aptamer 1.12.2—see Figure 3D.

The obtained results demonstrate that the equilibrium time for the inhibition of the aptamer–ligand interaction by ssDNA is determined by the location of the complementary site at the G-quadruplex (slow reactions) or the tail (rapid reactions). The length of the overlap between ssDNAs and the G-quadruplex does not influence the equilibrium time.

### 2.4. Influence of ssDNAs on Labeled OTA Binding with Aptamer

Further, the ssDNAs’ influence on aptamer–OTA binding was characterized under equilibrium conditions. For this purpose, we determined the dissociation constants of the aptamer–ssDNAs interaction in the presence of OTA-Flu. The earlier-developed protocol [26] was applied for this purpose. Firstly, the dissociation constant of the aptamer with OTA-Flu was determined. It was 183 ± 14 nM: see Figure S1 and Table S2. Then, the aptamer interacted with OTA-Flu in the presence of different concentrations of ssDNAs, and after that, the 50% inhibition point was determined. After this, the aptamer–ssDNA constant was determined using the known aptamer–OTA-Flu constant. Thus, the dependences of the percentage of bound OTA-Flu (F_bound_) on the concentration for 21 complementary ssDNAs or the unlabeled OTA were obtained.

**Figure 4:**
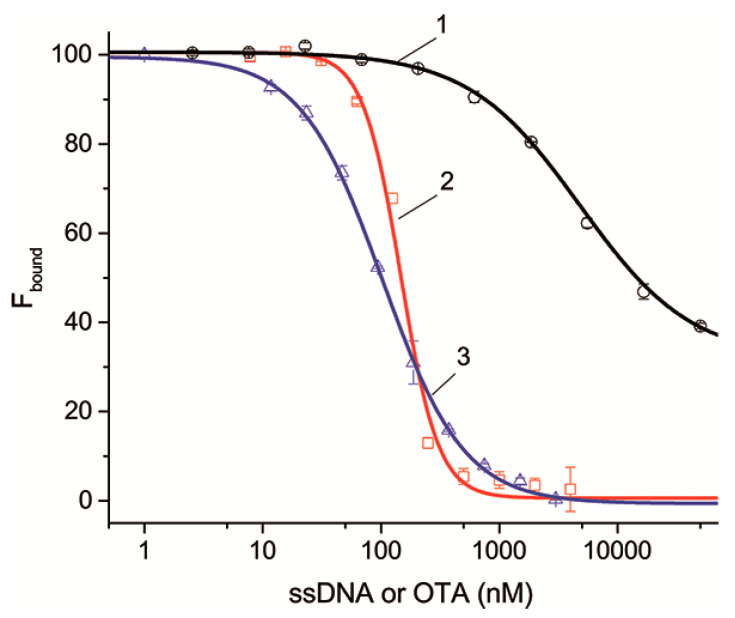
The dependences of the percentage of bound OTA-Flu on the concentration of ssDNA or OTA, (1)—{30–36}, (2)—{5–13}, and (3)—OTA. *n* = 3.

Figure 4 shows the dependences for ssDNAs {5–13} and {30–36} as examples characterized by different dissociation constants that are complementary to different structural elements of the aptamer. It can be seen that the dependences as well as the competitive interaction between OTA and OTA-Flu accord to the sigmoidal dependence. The complete set of inhibition curves for all ssDNAs is given in Figure S3. The parameters characterizing the inhibition of the aptamer–OTA-Flu interaction in the presence of ssDNAs are presented in Table 4.

Table 4 shows that an excess of ssDNA and OTA leads to the complete inactivation of the formation of the OTA-Flu complex with the aptamer, except for ssDNAs {30–36}, for which the degree of inactivation is 69%. This result is explained by the fact that ssDNAs {30–36} are complementary to six nucleotides of the 3’-tail, three of which do not influence them, with the remaining three having little influence on the binding of the aptamer to OTA (see Table S2). Thus, the dissociation constants for all ssDNAs have been determined. In this experiment, the constants also decrease with the increase in the number of H-bonds. 

The two obtained sets of dissociation constants of the aptamer–ssDNA interaction in the absence and presence of OTA allow for the characteration of the relationships between ssDNA features and their influence on aptamer–ligand binding.

### 2.5. Relations between ssDNAs Parameters and Inhibition of Ligand-Binding Activity Based on ITC and FA Data

**Figure 5:**
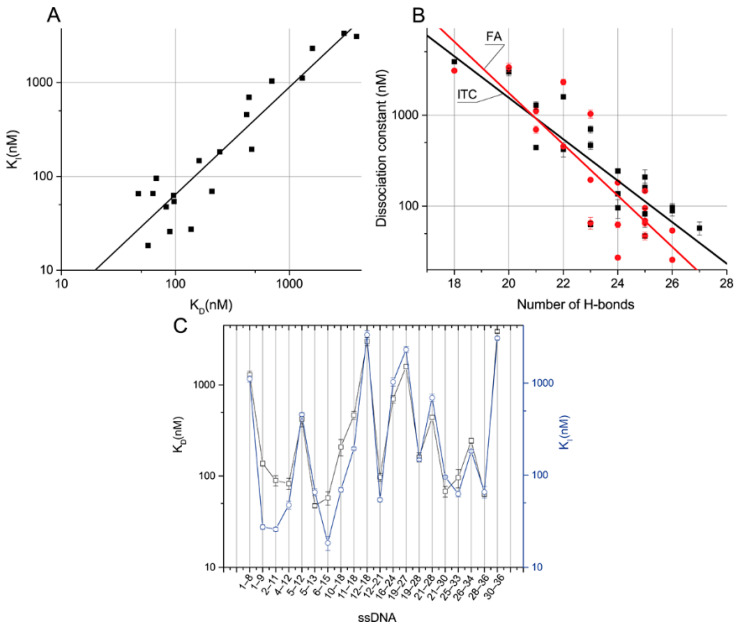
Dissociation constants of aptamer–ssDNA determined via OTA-Flu inhibition plotted versus the dissociation constants measured by direct interaction (adjusted R^2^ = 0.87) (**A**). Correlation of K_D_ (black) or K_I_ (red) with the number of hydrogen bonds; adjusted R^2^ is 0.75 and 0.76, respectively (**B**). Influence of the location of the complementary site on K_D_ (square) and K_I_ (circle) (**C**).

The obtained two sets of equilibrium constants of the aptamer–ssDNA dissociation (see Table 2 and Table 4) were compared. Figure 5A demonstrates that for all ssDNAs, they are close to each other and have the same order. This suggests a correlation between the dissociation constant of the aptamer–ssDNA complex and this constant calculated in the presence of OTA-Flu, reflecting the ability of ssDNA to inhibit the binding of aptamer 1.12.2 to the labeled OTA. The dependences of the obtained constants on the number of H-bonds were plotted (see Figure 5B). The distributions of the obtained constants divided by the number of H-bonds from the location of the complementary site were plotted, as shown in Figure 5C.

The linear approximation of the dependence of the K_I_, determined through the inhibition of OTA-Flu binding, from the K_D_, determined through direct interaction, in logarithmic coordinates, gives the following equation—K_I_ = –0.48 + 1.14 × K_D_, with adjusted R^2^ = 0.87 (Figure 5A). It can be seen that the constants correlate well with each other. 

Linear approximation of the dependences of these constants on the number of H-bonds in semi-logarithmic coordinates gives the following equations—H-bonds =7.7 − 0.228 × K_D_, with adjusted R^2^ = 0.75, and H-bonds = 8.9 − 0.28 × K_I_, with adjusted R^2^ = 0.76, respectively (Figure 5B). Thus, both dissociation constants tend to increase as the number of H-bonds increases. The correlation is not absolute and this fact can be explained by the nearest-neighbor effect [27], which consists of the influence of neighboring nucleotides on the formation of nucleotide pairs in DNA duplexes. It was noted in [28] that this effect is especially pronounced for sequences with a length of fewer than 12 nucleotides. Of all the characterized ssDNAs, the maximum degree of difference in K_D_ and K_I_ was observed for ssDNAs {28–26} and {16–24} with 23 H-bonds each, amounting to 11 and 16 times, respectively. The following data demonstrate a comparison of the aptamer–ssDNA dissociation constants with the aptamer–OTA one. 

Compounds from the ssDNA panel {1–9}, {2–11}, {4–12}, {5–13}, {6–15}, {12–21}, {21–30}, {25- 33}, and {28–36} have higher K_D_ than the aptamer–OTA constant, up to a maximum of 2.7 times. These ssDNAs are characterized by their number of H-bonds, ranging from 23 to 27. ssDNAs {1–9}, {2–11}, and {6–15} have higher K_I_ than the OTA, up to a maximum of two times the amount. These ssDNAs have a number of H-bonds in the range of 24 to 27. The remaining ssDNAs from the panel are characterized by lower constants, forming from 18 to 22 H-bonds with the aptamer.

No direct dependence of the binding constants on the location site was observed. Nevertheless, it should be noted that the difference between the K_D_ and K_I_ constants is most pronounced for ssDNAs whose binding sites are located at the 5’-end of the aptamer (see Figure 5C).

Almost all the tested ssDNAs affected the binding of the aptamer to the ligand, except for ssDNAs {1–5}, having a K_D_ less than 10^−5^ M and 13 H-bonds. The question remained open: how many H-bonds are sufficient to affect the aptamer–OTA binding? For this, in addition to the studied sequences, we selected three more complementary ssDNAs, {18–25}, {24–28}, and {28–33}, with 20, 14, and 15 H-bonds, respectively (see Table S3). In accordance with Section 3.4, their ability to inhibit the binding of the aptamer-labeled ligand was studied. Thus, none of the sequences affected the binding of the aptamer to the ligand (Figure S4). The results obtained make it possible to identify the range from 18 to 20 H-bonds as the limit below which ssDNA loses the ability to influence the binding of this aptamer to OTA.

### 2.6. Relationship between Inhibition Kinetics and Unfolding of Aptamer G-Quadruplex

According to the registered kinetic changes of FAs, in some cases, a long-term transition from an OTA-Flu–aptamer complex to a ssDNA complex was observed, which, according to [29,30], can be explained by the necessity of the unfolding of the G-quadruplex’s structure before aptamer–ssDNA complex formation. It was reasonable to confirm the relationship between the time of this transition and the structure of the G-quadruplex. The CD method was chosen for direct registration of structural changes in the aptamer. The method is convenient for studying G-quadruplexes since they have peculiar extrema that are not manifested by other DNA structures [31].

**Figure 6:**
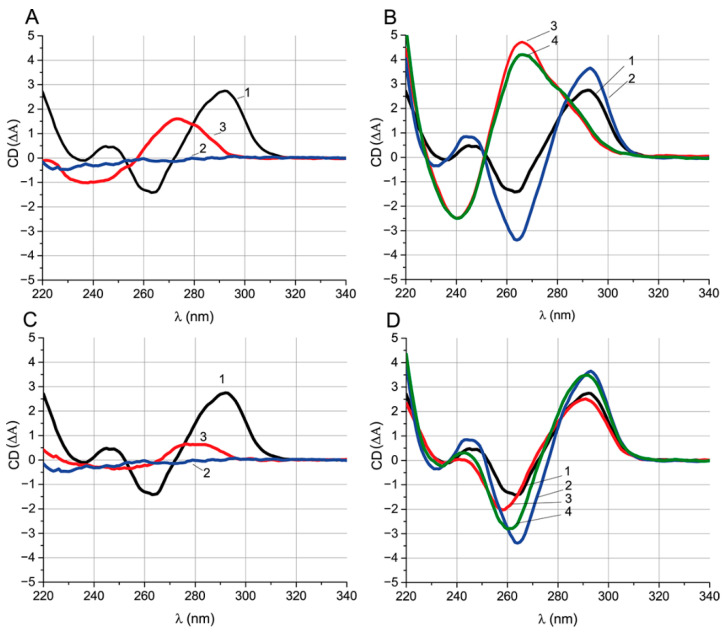
(**A**) CD spectra of aptamer (1), ochratoxin A (2), and ssDNA {6–15} (3). (**B**) CD spectra of the aptamer (1), the aptamer–OTA complex (2), the aptamer–ssDNA complex {6–15} (3), and the aptamer–OTA complex after the addition of ssDNA {6–15} (4). (**C**) CD spectra of aptamer (1), ochratoxin A (2), and ssDNA {28–36} (3). (**D**) CD spectra of aptamer (1), aptamer–OTA (2), aptamer–ssDNA {28–36} (3) and aptamer–ssDNA {28–36} after the addition of OTA (4). The reagent concentrations are 1 μM for all spectra.

For the experiment, two ssDNA were taken—{6–15} (group III) and {28–36} (group V), located in the G-quadruplex and 3’-tail, respectively. They have different equilibrium times of inhibition (less than a minute or more than 20 min, respectively), but similar K_D_ and K_I_ values that are close to the aptamer–OTA dissociation constant (see Table 2 and Table 4). CD spectroscopy was carried out for the aptamer, ochratoxin A, ssDNAs {6–15} and {28–36}, and their complexes (Figure 6). Their characteristic CD extrema were summarized in Table 5.

Figure 6A,C show the spectra of the primary components of the complexes. The spectrum of the aptamer has extrema typical of an antiparallel G-quadruplex [31] (see Table 5). One μM OTA has no pronounced extrema in the CD spectrum and is at the background level over the entire studied wavelength range. ssDNAs {6–15} and {28–36} have one positive and one negative extremum each; the position of the extrema is typical for ssDNA. The position of the ssDNA extrema in the CD spectra depends on the nucleotide composition [31]. Therefore, the values of the extrema for ssDNA {6–15} and {28–36} are different. We used the CD and absorption spectra (Figure S5) of the initial reagents for the correct interpretation of the CD spectra of their complexes.

Figure 6B compares spectra of the aptamer and the aptamer–{6–15}, aptamer–OTA, and aptamer–OTA complexes after the addition of ssDNA {6–15}. The absorption spectra of these complexes are shown in Figure S5A. Comparison of the CD spectra of the aptamer and aptamer–OTA shows that the interaction with OTA increases the amplitudes of all G-quadruplex extrema of aptamer and is accompanied by extrema shifts (see Table 5). The interaction takes less than 3 min (Figure S6A). Since the OTA that under-considered concentration does not have its own CD spectral peaks, it can be unequivocally stated that the change in the aptamer spectrum is associated with the formation of the complex, and not with the superposition of the OTA spectrum.

The addition of ssDNA {6–15} to the aptamer leads to the loss of the extrema typical for the G-quadruplex (see Figure 6B). The unfolding of the G-quadruplex takes about 10 min in the absence of OTA (Figure S6B), which is less than was previously (see Section 2.3) observed in FA experiments. The resulting spectrum has extrema typical for double-stranded DNA [32]. In order to observe the influence of the presence of OTA on the unfolding of the quadruplex during its interaction with ssDNA, the aptamer was preincubated with OTA, and then ssDNA was added. In the presence of OTA, the complete unfolding of the G-quadruplex takes about 70 min, seven times more than in OTA’s absence (Figure S6B). Comparison of the CD equilibrium spectra for the aptamer–{6–15} and aptamer–OTA–{6–15} systems under equilibrium conditions shows that they are practically identical regardless of OTA: in both cases, the G-quadruplex is unfolded. No traces of the aptamer–OTA complex were observed in the aptamer–OTA–ssDNA {6–15} system.

A similar comparison of the equilibrium CD spectra of the complexes for ssDNA {28–36} were carried out (Figure 6D). The aptamer–ssDNA complex {28–36} retains the extrema typical for the antiparallel G-quadruplex and is accompanied only by the shift in the spectra in the range of 240–260 nm down (see Figure 6D). Formation of the complex takes less than 2 min (Figure S6A). In contrast to experiments with ssDNA {6–15}, the aptamer was preincubated with ssDNA rather than OTA to hinder the aptamer–OTA interaction. The spectrum of the aptamer–ssDNA complex {28–36} after the addition of OTA was typical for a G-quadruplex being the intermediate between the spectra of the aptamer–ssDNA and the aptamer–OTA complexes. An observed decrease in the CD value at 260 nm and increase at 290 nm indicates the formation of an aptamer–OTA complex (Figure 6D). 

These examples of studies on ssDNAs {6–15} and {28–36} demonstrate that the long kinetics of an aptamer transition between its complexes with OTA and ssDNA is related with the G-quadruplex unfolding under ssDNAs’ influence and is observed in cases when ssDNA overlaps with the G-quadruplex. However, the observed long duration cannot be explained by unfolding time alone, so the presence of the stabilization effect of OTA on the aptamer’s G-quadruplex stability is highly certain and leads to significantly prolonged G-quadruplex unfolding in OTA’s presence. 

A specific feature of ssDNAs from groups II and IV is their complementarity to both the quadruplex and tail of the aptamer. The confirmation of the retention or absence of OTA binding for these ssDNAs would allow for estimating the necessity of the quadruplex structure for OTA binding.

### 2.7. Refolding of Aptamer G-Qaudruplex from Aptamer–ssDNA Complex in Presence of OTA

**Figure 7:**
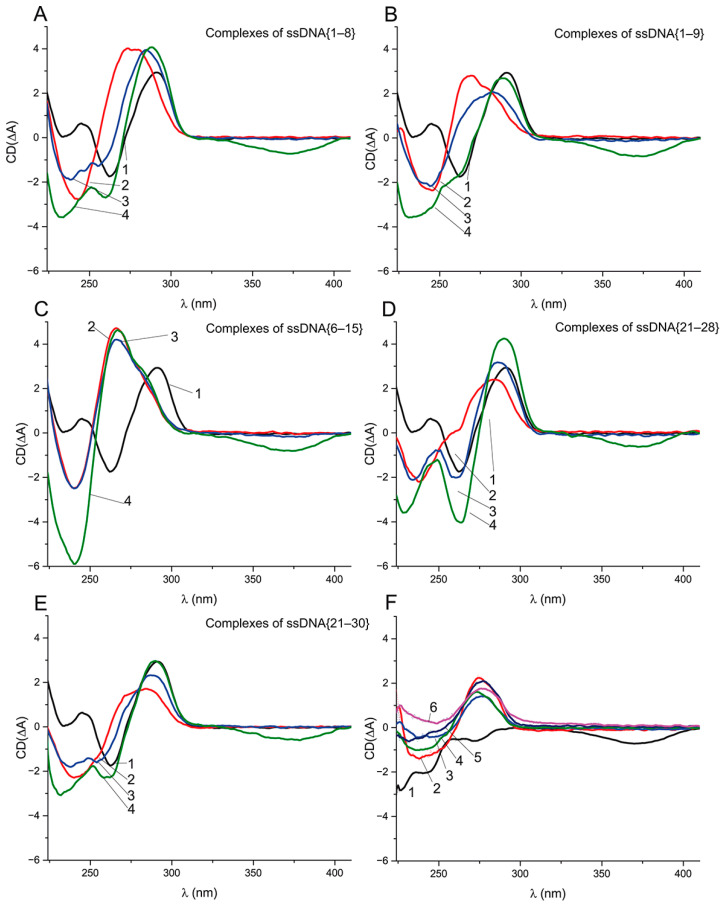
CD spectra of 1 μM aptamer (1) and its complexes with ssDNA (2) and with ssDNA after addition of 1 μM (3) and 10 μM (4) of OTA—(**A**) 2 μM ssDNA {1–8}; (**B**) 1 μM ssDNA {1–9}; (**C**) 1 μM ssDNA {6–15}; (**D**) 1.5 μM ssDNA {21–28}; (**E**) 1 μM ssDNA {21–30}. (**F**) CD spectra of 10 μM ochratoxin A (1), 2 μM ssDNA {1–8} (2), 1 μM ssDNA {1–9} (3), 1 μM ssDNA {6–15} (4), 1.5 μM ssDNA {21–28} (5), and 1 μM ssDNA {21–30} (6).

To study the refolding of the G-quadruplex from the aptamer–ssDNA complex in the presence of OTA, we measured the CD spectra of the aptamer complexes with ssDNA and with ssDNA in the presence of OTA at equal (1 μM) and excess (10 μM) molar concentrations, relative to the aptamer concentration. Besides ssDNA {6–15}, whose complementary site is entirely localized in the G-quadruplex, we chose four ssDNAs ({1–8}, {1–9}, {21–28}, and {21–30}) that overlap with not only the G-quadruplex, but also the 5’ or 3’ tail. The chosen ssDNAs {1–9} and {21–30} has KD values of about 60 nM, being similar to ssDNA {6–15}. In contrast, ssDNAs {1–8} and {21–28} have KD 1290 ± 118 and 441 ± 22 nM, respectively. In all four cases, the unfolding times of the G-quadruplex during the reaction with ssDNA were about 10 min, being similar to {5–16}, and the refolding in the presence of OTA takes about 70 min. The obtained CD spectra of all the complexes are given in Figure 7.

Figure 7A,B, indicate the unfolding of the G-quadruplex caused by ssDNAs {1–8} and {1–9} and the formation of dsDNA. For both ssDNAs in the presence of 1 μM of OTA, the spectrum (3) shows the shift in the positive extrema from 275 to 285 nm, i.e., to the initial location of the extremum for the G-quadruplex (Figure 7A,B). The shift is more pronounced in the case of ssDNA {1–8}. Note that three extrema in the range 230–260 nm are typical of the CD of an antiparallel quadruplex (Figure 7A). The addition of the excess (10 μM) of OTA (see spectra 4 in Figure 7) leads to the further transition of the aptamer–ssDNA spectra into the spectra similar to the antiparallel G-quadruplex (Figure 7A,B). That gives us evidence that the aptamer preserves OTA-binding activity in complexes with ssDNAs {1–8} and {1–9}. The transition from the aptamer–ssDNA complex to the aptamer–OTA complex is accompanied by the restoration of the initial quadruplex structure. Interestingly, in the case of an aptamer complex with ssDNA {6–15}, no changes occurred, not only in the case of the equimolar OTA:aptamer ratio but also for a 10-fold excess of OTA (see Figure 7C). The complexes of aptamer with ssDNAs {21–28} and {21–30} in the presence and absence of OTA behaved similarly as the complexes with ssDNAs {1–8} and {1–9} (Figure 7D,E). The CD spectral extrema of all complexes are presented in Table S5.

The comparison of aptamer–ssDNA complexes with the same K_D_ (Figure S7) shows the similarity of extrema between ssDNAs {1–9} and {5–16}, which partly overlap with each other, but for ssDNA {21–30}, the positive extremum is noticeably different. Therefore, the complexes in which the quadruplex was unfolded from different ends had different spectra (see Figure 7B,E), suggesting structural differences between them. The comparison of aptamer–ssDNA complexes in the presence of OTA excess showed that, unexpectedly, the spectra with the most pronounced G-quadruplex extrema were observed in the case of ssDNA {21–30}, whereas the least affinity to the aptamer was demonstrated for ssDNA {1–8}. Thus, for all five ssDNAs, the interaction with the aptamer led to its unfolding, whereas only ssDNA {5–16} caused the loss of the OTA-binding ability. The following differences of the studied ssDNAs by the location of the quadruplex’s overlapping could be marked:

(1) ssDNAs {1–8} and {1–9} have five bases with an overlapping 5′-aptamer tail and three or four bases overlapping the quadruplex, respectively, including the sixth and seventh guanines, which participate in the formation of both guanine tetrads of the G-quadruplex.
(2) The aptamer’s site complementary to ssDNA {6–15} consisted of 10 bases entirely localized in the quadruplex region and overlapping with the 6th, 7th, 11th, 12th, and 15th guanines participating in both guanine tetrads’ formation.
(3) ssDNAs {21–28} and {21–30} have 4 and 6 bases overlapping with the 3′-tail, respectively, and 4 bases overlapping the quadruplex, including the 23th and 24th guanines participating in the guanine tetrads’ formation.

Therefore, the aptamer–ssDNA complexes with the ssDNAs that overlap the quadruplex from the 3′-tail (Figure 7D,E) shown in the OTA’s presence have a higher similarity of their CD spectra with the antiparallel quadruplex than the ssDNAs with the overlap location at the 5′-tail (Figure 7A,B) despite the close quantity of H-bonds (11 vs. 10) with the quadruplex region. Our data confirm that OTA-binding properties can be restored even after the unfolding of the G-quadruplex, as the CD data for ssDNAs {1–8}, {1–9}, {21–28}, and {21–30} demonstrated.

It is known that a number of low-molecular-weight compounds are capable of initiating the rearrangement of the aptamer from a duplex to a quadruplex [33]. We demonstrated that in the case of OTA, the relation between the refolding of the aptamer quadruplex from a duplex in the presence of OTA is complicated and depends on the location and length of the overlap between the complementary site of the ssDNA and the quadruplex region of the aptamer.

### 2.8. Prospects of Uses of the Described ssDNA-Induced Effects on the Aptamer–OTA Binding in Aptasensors

The results of the conducted experiments show that an ssDNA needs to have a specific number of H-bonds to influence the aptame–ligand binding ability. For anti-OTA aptamer 1.12.2, the minimal required number of H-bonds is ranged from 18 to 20. The influence of ssDNAs on the aptamer-binding ability and/or its structure can be applied to produce OTA-dependent signals, namely:

- OTA-induced ssDNA displacement, accompanied by a switch in the aptamer’s structure from a dsDNA complex to a G-quadruplex (ssDNAs {1–8}, {1–9}, {21–28}, {21–30});
- OTA-induced change of the G-quadruplex’s unfolding time under complexation with ssDNA (ssDNA {5–16});
- OTA-induced ssDNA displacement without structural rearrangement (ssDNA {28–36}).

These effects can be used to develop aptasensors for OTA because of (1) the degree of inhibition of the aptamer–ligand interaction is directly proportional to the number of H-bonds; (2) the process of ssDNA displacement in the presence of OTA is reversible. However, the processes of the duplex-to-quadruplex switch were found to be prolonged (from 20 (FA data) to 70 (CD data) min); this feature limits analytical applications. Therefore, the rapid displacement of ssDNA (without G-quadruplex transformation, such as in the case of ssDNA {28–36}) seems to be preferable for aptasensors. It will extend the row of aptasensors and will realize approaches differing from the known works where the ssDNA’s complementary solely to the 3’-tail caused OTA-induced ssDNA displacement [34,35,36].

## 3. Materials and Methods

### 3.1. Reagents and Sample Preparation

The OTA powder was obtained from Fermentek (Israel). The lyophilized DNA aptamer (5′-GAT-CGG-GTG-TGG-GTG-GCG-TAA-AGG-GAG-CAT-CGG-ACA-3′) and its complementary ssDNAs (see Table 1) were custom-synthesized and purified by Syntol (Russia). The ssDNAs were designed using the Multiple Primer Analyzer (Thermofisher Scientific, USA, MA, Waltham) and OligoCalc (http://biotools.nubic.northwestern.edu/OligoCalc.html accessed on 22 April 2022).

Tris(hydroxymethyl)aminomethane, dimethyl sulfoxide (DMSO), and sodium acetate were obtained from Sigma-Aldrich (USA, MO, St. Louis), 4’-aminomethyl fluorescein—from Thermo Fisher Scientific (USA, MA, Waltham), magnesium chloride—from Honeywell (USA, NC, Charlotte) and calcium chloride—from Scharlau (Spain, Catalonia, Barcelona). All chemicals were of analytical or reagent grade. 

A Simplicity Milli-Q^®^ system from Millipore (USA, MA, Burlington) was used to obtain ultrapure water for buffers and reagent solutions. Stock solutions of the aptamer and oligonucleotides were prepared by dissolving lyophilized DNA in deionized water to the concentration of 400 μM. A NanoDrop2000 microvolume spectrophotometer (Thermo Scientific, USA, MA, Waltham) was used for verification of the concentrations of the aptamer and oligonucleotides.

An OTA stock solution was prepared by dissolving 1 mg of OTA in 100 μL of DMSO to the concentration of 24.7 mM. Then, it was diluted in mQ water to the concentration of 400 μM. OTA labeled with 4’-aminomethyl fluorescein was synthesized using the protocol described previously [26,37]. It was diluted in methanol and stored at 4 °C in the dark.

### 3.2. Characterization of Aptamer–ssDNA and Aptamer–OTA Interactions Using Isothermal Titration Calorimetry

Isothermal titration calorimetry was performed using the MicroCal PEAQ-ITC (Malvern Panalytical, UK, Worcestershire, Malvern). All interactions were carried out in WB buffer (20 mM Tris-acetate buffer, pH = 8.4, containing 100 mM sodium acetate, 10 mM MgCl_2_, 10 mM CaCl_2_) at 25 °C. Binding experiments were performed for four combinations of the aptamer and ssDNA concentrations: (1) 4 μM and 50 μM; (2) 8 μM and 100 μM; (3) 20 μM and 200 μM; and (4) 25 μM and 300 μM, respectively. Additionally, ITC was used to characterize the aptamer (5 μM) and OTA (50 μM) pair using the same protocol.

All samples were degassed before use. At first, 280 μL of the aptamer was added into the reaction cell, and then the titration syringe with 30 μL of ssDNA or OTA was set up. Binding experiments typically consisted of 14 successive 2.4 μL injections (except for the first 0.4 μL injection) of ssDNA every 180 s with constant stirring at 750 rpm. To exclude thermal effects associated with dilution, control experiments with WB instead of the aptamer were implemented, and the values obtained were used to correct the raw ITC data for specific interactions.

The equilibrium dissociation constant (K_D_), stoichiometry (N), and enthalpy (∆H) were determined by multiparametric approximation by the MicroCal PEAQ-ITC Analysis Software using the one-site binding model. Then, the Gibbs energy (∆G) and the entropy (∆S) were calculated. 

### 3.3. Kinetics of Aptamer–ssDNA Interactions Followed by Fluorescence Anisotropy of the OTA-Flu Probe

All fluorescence anisotropy (FA) measurements were performed in black non-binding 96-well microplates from Thermo Scientific NUNC^TM^ (Denmark, Roskilde) using a CLARIOstar (BMGLabtech, Germany, Baden-Wurttemberg, Ortenberg) multimode plate reader with an excitation filter (490 + 10 nm), dichroic mirror (504 nm), and emission filter (520 + 10 nm).

Solutions of ssDNAs were prepared with the following concentrations: 100 µM for ssDNAs {1–5}, {16–24}, {30–36}, {19–27}; 72 µM for ssDNAs {1–8}, {12–18}, {21–28}; 40 µM for ssDNA {26–34}, 20 µM for ssDNA {26–34}, and 4 µM for the rest of the others. Then 100 µL of ssDNA, 50 µL of 20 nM OTA-Flu solution in WB, and 50 µL of 360 nM aptamer solution were successively added to microplate wells, and FA values were continuously registered during the 35 min. Time delay before the first measurement in all cases was no more than one minute. The mixtures (100 µL WB + 50 µL OTA-Flu + 50 µL aptamer) and (50 µL OTA-Flu + 150 µL WB) were used to determine the experimental window (i.e., the maximum and the minimum FA values, respectively).

The data were analyzed using the CLARIOstar MARS software and the exponential decay fittings (y = y_0_ + Aε^−x/t^) were calculated using Origin 8.1 (Origin Lab, USA, MA, Northampton). The obtained time dependences of FA were transformed to the time dependences of the bound OTA-Flu fraction (F_bound_) as described earlier [26].

### 3.4. Characterization of Aptamer–ssDNA Interactions by Fluorescence Anisotropy of the OTA-Flu Probe at Equilibrium

Constants of the interactions of 21 ssDNAs with the aptamer were measured through the inhibition of aptamer–OTA-Flu complex formation by the FA method and calculated as described below [26].

Firstly, the saturation experiment was carried out to calculate the dissociation constant between the aptamer and the labeled OTA. More specifically, the 19 µM aptamer stock solution in WB and 10 successive 3-fold dilutions thereof were prepared. Then, 100 µL of these dilutions and 100 µL of OTA-Flu (10 nM) were added to microplate wells. The microplate was softly shaken for 2 min at room temperature, and FA values were measured. Taking into account the fluorescence change of OTA-Flu, fractions of bound OTA-Flu (F_bound_) were calculated, where F_bound_ = 0 was the FA of free OTA-Flu, and F_bound_ = 100 was the FA of its bound state.

Then competitive experiments for each ssDNA were carried out. The same ssDNA solutions as in Section 3.3 were used. A total of 10 successive 3-fold dilutions of 21 ssDNAs and of OTA were prepared. Like in the previous experiment, 100 µL of diluted ssDNA or OTA was added to each microplate well. Then, 50 µL of 20 nM OTA-Flu solution was added in WB, and 50 µL of 360 nM aptamer solution was added to the wells. After 40 min, FA values were measured. Twenty-two dependences of F_bound_ from the OTA and ssDNA’s concentrations were obtained. The data were approximated by the sigmoidal function (y = A_2_ + ((A_1_ − A_2_)/(1 + (x/x_0_)^p^)) using Origin 8.1.

### 3.5. Circular Dichroism Spectra Measurements at Equilibrium

All measurements were carried out using a Chirascan spectrometer (Applied Photophysics, UK, Surrey, Leatherhead) and a 1.5 nm bandwidth and 2 ns integration time. The CD and absorbance spectra of the aptamer, OTA, ssDNAs, and their complexes in WB were obtained at room temperature in the range from 220 to 420 nm. CD was measured as the differential absorbance (ΔA) [mdeg] of left (A_LCP_) and right circularly polarized (A_RCP_) light:

ΔA= A_LCP_ − A_RCP_. (1)

First, 4 μM stock solutions of aptamer, OTA, and ssDNA in WB were prepared. In case of ssDNAs {1–8} and{21–28}, the concentration was 8 and 6 μM respectively. CD spectra in equilibrium conditions were measured using the following mixtures. (1) Individual reagents were four-fold diluted in WB to a final concentration of 1μM. (2) In the case of aptamer–OTA or aptamer–ssDNA complexes, 220 μL of the aptamer was mixed with an equal volume of ssDNA (or OTA) and diluted with 440 μL of WB. (3) In the case of aptamer–OTA–ssDNA {6–15} interactions, 220 μL of aptamer and OTA were first mixed in WB. After 15 min, the ssDNA {6–15} was added up to a final volume of 880 μL. (4) In the case of aptamer–ssDNA {28–36}–OTA mixture, firstly, equal volumes of aptamer, ssDNA, and WB were mixed into the volume of 660 μL. After 15 min, 220 μL of OTA was added. Thus, for all studies, the molar ratio reached was either 1:1 or 1:1:1.

To study G-quadruplex unfolding in the presence of different ssDNAs, 40 μM OTA stock solution was prepared. Then, spectra of 10 μM OTA, 2 μM ssDNA {1–8}, 1 μM ssDNA {1–9}, 1.5 μM ssDNA {21–28}, and (5) 1 μM ssDNA {21–30} and complexes of aptamer–ssDNA were obtained as described above. After that, the mixtures of 220 μL of 4 μM aptamer, 220 μL of the ssDNA, and 220 μL of WB were incubated for 30 min, then 220 μL of OTA in concentrations of 1 or 10 μM was added and incubated for 80 min.

The CD data were analyzed using the Pro-Data Chirascan package (Applied Photophysics, UK) and processed using Origin 8.1 (Origin Lab, USA).

## 4. Conclusions

For the first time, the influence of complementary ssDNA on the aptamer’s G-quadruplex structure and ligand-binding are considered by using a consecutive ssDNA row (21) with different lengths and locations relative to complementary regions. For a row of 21 ssDNAs fully complementary to the anti-OTA aptamer (1.12.2), the number of hydrogen bonds forming between ssDNA and the aptamer affected the inhibition efficiency of the OTA binding, and the position of the complementary region affected the inactivation kinetics. The obtained results demonstrated the correlation between two sets of aptamer–ssDNA dissociation constants that were calculated directly by ITC and indirectly through ssDNA inactivation of aptamer-labeled OTA binding by FA (R^2^ = 0.87). The ssDNAs having more than 23 hydrogen bonds with the aptamer have a lower aptamer dissociation constant than for aptamer–OTA interactions (131 ± 32 and 36.3 ± 2.1 nM, respectively). The ssDNAs having less than 18 hydrogen bonds did not affect the aptamer–OTA affinity 

The complete unfolding of the quadruplex in the OTA’s absence occurred within 10 min. In the OTA’s presence, the time for both the unfolding and refolding of the G-quadruplex was seven-fold longer. These results confirm the OTA-mediated G-quadruplex’s stabilization. They demonstrated that the location of ssDNA relative to the quadruplex influences the retention or loss of OTA-binding properties. In particular:

- The refolding of the G-quadruplex’s structure in the presence of OTA after the aptamer–ssDNA binding occurs if the ssDNA interacts with 4–5 bases of the G-quadruplex and some bases from 5’- or 3’-tail;
- The location of the complementary site of ssDNA in the quadruplex coupled with high (such as 27) quantity of H-bonds leads to the loss of OTA-binding properties;
- The location of the complementary site in the aptamer’s tail did not affect the structure of the G-quadruplex, and such ssDNAs were rapidly displaced by OTA from their complex with aptamer.

Although the significant structural variability of aptamers and the ligands undoubtedly affects the modulation of aptamer–ligand activity via complementary oligonucleotides, the considered study demonstrated relations between the possible modulation effects and parameters of ssDNAs. These results should be considered in the development of ssDNA-based aptamer sensors and molecular tools. 

## Figures and Tables

**Table 1:** The selected panel of complementary ssDNAs to study the inhibition of the ligand-binding ability of the anti-OTA aptamer 1.12.2.

Binding Site	Sequences from 5’ to 3’ of the Aptamer and ssDNAs																																				Purines	Pyrimidines	Length	H-Bonds
	G	A	T	C	G	G	G	T	G	T	G	G	G	T	G	G	C	G	T	A	A	A	G	G	G	A	G	C	A	T	C	G	G	A	C	A				
	1	2	3	4	5	6	7	8	9	10	11	12	13	14	15	16	17	18	19	20	21	22	23	24	25	26	27	28	29	30	31	32	33	34	35	36				
{1–5}	C	G	A	T	C																																3	2	5	13
{1–8}	A	C	C	C	G	A	T	C																													5	3	8	21
{1–9}	C	A	C	C	C	G	A	T	C																												6	3	9	24
{2–11}		C	A	C	A	C	C	C	G	A	T																										6	4	10	26
{4–12}				C	C	A	C	A	C	C	C	G																									7	2	9	25
{5–12}					C	C	A	C	A	C	C	C																									6	2	8	22
{5–13}					C	C	C	A	C	A	C	C	C																								7	2	9	25
{6–15}						C	A	C	C	C	A	C	A	C	C																						7	3	10	27
{10–18}										C	G	C	C	A	C	C	C	A																			7	2	9	25
{11–18}											C	G	C	C	A	C	C	C																			7	1	8	23
{12–18}												C	G	C	C	A	C	C																			6	1	7	20
{12–21}												T	T	A	C	G	C	C	A	C	C																6	4	10	26
{16–24}																C	C	T	T	T	A	C	G	C													5	4	9	23
{19–27}																			C	T	C	C	C	T	T	T	A										4	5	9	22
{19–28}																			G	C	T	C	C	C	T	T	T	A									5	5	10	25
{21–28}																					G	C	T	C	C	C	T	T									5	3	8	21
{21–30}																					A	T	G	C	T	C	C	C	T	T							5	5	10	25
{25–33}																									C	C	G	A	T	G	C	T	C				6	3	9	24
{26–34}																										T	C	C	G	A	T	G	C	T			6	3	9	24
{28–36}																												T	G	T	C	C	G	A	T	G	5	4	9	23
{30–36}																														T	G	T	C	C	G	A	4	3	7	18

These sequences can be divided into five groups based on the aptamer region they overlay. I: One ssDNA {1–5} complementary to the 5’-tail of the aptamer. II: Six ssDNAs that overlap with the 5’-tail and part of the G-quadruplex of the aptamer—{1–8}, {1–9}, {2–11}, {4–12}, {5–12}, {5–13}. III: Six ssDNAs that are only complementary to the G-quadruplex of the aptamer—{6–15}, {10–18}, {11–18}, {12–18}, {12–21}, {16–24}. IV: Five ssDNAs that overlap with the G-quadruplex and 3’-tail of the aptamer—{19–27}, {19–28}, {21–28}, {21–30}, {25–33}. V: Three ssDNAs that bind with the 3’-tail region—{26–34}, {28–36}, {30–36}. It should be noted that for two ssDNAs, ({28–36} and {30–36}), the complementary regions contain three terminal 3’-nucleotides that do not affect OTA binding.

**Table 2:** Thermodynamic parameters of the interaction of the aptamer with ssDNAs and OTA.

Binding Site	H-Bonds	Length (Bases)	K_D_ (nM)	∆H (kcal/mol)	∆S∙T (kcal/mol)∙K	∆G (kcal/mol)	N Sites
{1–5}	13	5	>10000	−3.24	−3.01	−6.25	1.00
{1–8}	21	8	1290 ± 118	−53.7	45.6	−8.04	1.05
{1–9}	24	9	137 ± 9	−65.8	56.5	−9.37	1.07
{2–11}	26	10	89.4 ± 11.4	−63.7	54.0	−9.63	1.00
{4–12}	25	9	82.8 ± 11.6	−52.8	43.1	−9.67	1.08
{5–12}	22	8	421 ± 75	−42.0	33.3	−8.70	1.01
{5–13}	25	9	47.2 ± 2.3	−61.8	51.8	−10.0	1.03
{6–15}	27	10	57.5 ± 9.6	−56.8	46.8	−9.88	0.98
{10–18}	25	9	182 ± 37	−49.0	49.1	−9.20	1.02
{11–18}	23	8	466 ± 46	−42.1	33.5	−8.64	0.95
{12–18}	20	7	3002 ± 297	−28.5	21.0	−7.53	1.06
{12–21}	26	10	97.2 ± 9.2	−50.3	40.7	−9.57	1.04
{16–24}	23	9	701 ± 65	−42.2	33.8	−8.50	0.97
{19–27}	22	9	1590 ± 86	−45.8	37.9	−7.91	0.93
{19–28}	25	10	161 ± 19	−42.2	33.0	−9.27	1.01
{21–28}	21	8	441 ± 22	−35.6	26.9	−8.67	1.04
{21–30}	25	10	68 ± 9	−49.7	39.9	−9.78	0.99
{25–33}	24	9	96.1 ± 22.1	−44.2	34.6	−9.58	0.93
{26–34}	24	9	244 ± 14	−45.9	36.9	−9.03	0.95
{28–36}	23	9	63.5 ± 4.2	−47.9	38.1	−9.82	0.91
{30–36}	18	7	3870 ± 153	−44.1	36.7	−7.39	0.91
Ochratoxin A			131 ± 32	−20.4	10.9	−9.39	1.05

**Table 3:** Kinetic parameters of OTA-Flu released from the complex with the aptamer caused by an excess of ssDNAs.

Binding Site	Group	H-Bonds	Half-Life (s)	Equilibrium Time (min)	Decay Rate (s^−1^)
{1–8}	II	21	267	29.7	2.59 × 10^−3^
{1–9}	II	24	345	38.3	2.01 × 10^−3^
{2–11}	II	26	356	39.5	1.94 × 10^−3^
{4–12}	II	25	349	38.7	1.98 × 10^−3^
{5–12}	II	22	184	20.4	3.75 × 10^−3^
{5–13}	II	25	344	38.1	2.01 × 10^−3^
{6–15}	III	27	333	37.0	2.08 × 10^−3^
{10–18}	III	25	325	36.0	2.13 × 10^−3^
{11–18}	III	23	202	22.4	3.43 × 10^−3^
{12–18}	III	20	192	21.2	3.61 × 10^−3^
{12–21}	III	26	359	39.7	1.93 × 10^−3^
{16–24}	III	23	281	31.1	2.47 × 10^−3^
{19–27}	IV	22	232	25.7	2.98 × 10^−3^
{19–28}	IV	25	208	23.0	3.33 × 10^−3^
{21–28}	IV	21	203	22.4	3.42 × 10^−3^
{21–30}	IV	25	361	39.3	1.92 × 10^−3^
{25–33}	IV *	24	-	>1	-
{26–34}	V	24	-	>1	-
{28–36}	V	23	-	>1	-
{30–36}	V	18	-	>1	-

* Data suggest assignment of this ssDNA to V group.

**Table 4:** Equilibrium parameters for the inhibition of the interaction of the aptamer with OTA-Flu by an excess of ssDNA.

Binding Site	H-Bonds	L_50_ (nM)	K_I_ (nM)	Inhibition Degree
{1–5}	13	NB *	-	-
{1–8}	21	1934 ± 142	1116 ± 85	99.4 ± 1.2
{1–9}	24	89.8 ± 2.3	27.3 ± 1.5	97.8 ± 0.9
{2–11}	26	87.6 ± 2.0	25.9 ± 1.3	97.1 ± 0.8
{4–12}	25	119.3 ± 6.9	47.4 ± 4.6	95.9 ± 1.3
{5–12}	22	722 ± 29	456.0 ± 19.9	99.7 ± 0.8
{5–13}	25	146.1 ± 8.2	65.5 ± 5.5	99.4 ± 2.7
{6–15}	27	76.6 ± 4.6	18.4 ± 3.1	98.0 ± 1.1
{10–18}	25	151.7 ± 5.7	69.3 ± 3.8	96.2 ± 0.3
{11–18}	23	336.9 ± 7.4	195.0 ± 5.0	99.5 ± 0.7
{12–18}	20	5678 ± 588	3362 ± 352	100.0 ± 2.5
{12–21}	26	129.3 ± 4.1	54.1 ± 2.8	97.7 ± 0.9
{16–24}	23	1577 ± 157	1036 ± 107	96.7 ± 2.2
{19–27}	22	3465 ± 253	2317 ± 172	99.8 ± 1.6
{19–28}	25	267.1 ± 6.5	147.6 ± 4.4	99.1 ± 0.6
{21–28}	21	1235 ± 105	696.4 ± 63.0	98.7 ± 1.4
{21–30}	25	190.5 ± 3.5	95.6 ± 2.4	98.1 ± 0.8
{25–33}	24	142.1 ± 6.8	62.8 ± 4.6	101.1 ± 1.9
{26–34}	24	318.5 ± 6.8	182.4 ± 4.6	98.3 ± 0.3
{28–36}	23	146.5 ± 14.3	65.7 ± 9.7	92.5 ± 0.6
{30–36}	18	4815 ± 345	3094 ± 133	68.2 ± 1.8
Ochratoxin A		106.2 ± 6.4	36.3 ± 2.1	100.6 ± 1.8

* NB—no binding detected.

**Table 5:** CD extrema for the aptamer, OTA, ssDNAs, and their complexes *.

Sample*	Extrema Wavelength (nm)			
	«−»	«+»	«−»	«+»
Aptamer	236	246	261	292
OTA	-	-	-	-
ssDNA {6–15}	237		273	
ssDNA {28–36}	250		283	
Aptamer + OTA	232	246	264	293
Aptamer + ssDNA {6–15}	240		268	
Aptamer + ssDNA {28–36}	235	243	259	292
Aptamer + OTA + ssDNA {6–15}	240		268	
Aptamer + ssDNA {28–36} + OTA	234	244	262	292

* All concentrations are equal to 1 μM.

## Data Availability

The data presented in this study are available on request from the corresponding author.

## Supplementary Materials

The following supporting information can be downloaded at: https://www.mdpi.com/article/10.3390/ijms23094876/s1.
