# Supplementary material for: Modulation of Aptamer–Ligand-Binding by Complementary Oligonucleotides: A G-Quadruplex Anti-Ochratoxin A Aptamer Case Study
Source: Int J Mol Sci. 2022 Apr 28;23(9):4876. doi: 10.3390/ijms23094876 (PMC9103105; doi:10.3390/ijms23094876)
Supplement: Supplementary file 1 [file ijms-23-04876-s001.zip › ijms-1678668-supplementary.pdf]

SUPPORTING INFORMATION

Modulation of Aptamer-Ligand-Binding by Complementary Oligonucleotides: a G-Quadruplex Anti-Ochratoxin A Aptamer Case Study

Alexey V. Samokhvalov[1], Irina V. Safenkova[1], Sergei A. Eremin[1,2], Artem N. Bonchuk[3] Oksana G. Maksimenko[3], Nikolai N. Sluchanko[1], Anatoly V. Zherdev[1], Boris B. Dzantiev[1*]

[1]A.N. Bach Institute of Biochemistry, Research Center of Biotechnology, Russian Academy of Sciences, Moscow 119071, Russia

[2]Faculty of Chemistry, M.V. Lomonosov Moscow State University, Moscow 119991, Russia

[3]Center for Precision Genome Editing and Genetic Technologies for Biomedicine, Institute of Gene Biology, Russian Academy of Sciences, Moscow 119334, Russia

*Correspondence: dzantiev@inbi.ras.ru; Tel.: +7-495-954-3142; Fax: +7-495-954-2804

S1 Measurements of interaction of aptamer and its shortened variants with labeled ligand (OTA-Flu) by fluorescence anisotropy.

To verify previous data considering influence of aptamer tail regions on OTA binding [16,19] we carry out fluorescent anisotropy measurement of interaction between 4´-aminomethyl fluorescein labeled OTA and aptamer or it shortened variants as mentioned in Section 2.4 of Materials and Methods, see Table s1.

Table S1 Sequences of aptamer and its shortened variants

Name	Sequence from 5' to 3'
Aptamer 1.12.2	gat cgg gtg tgg gtg gcg taa agg gag cat cgg aca
Mut1	_at cgg gtg tgg gtg gcg taa agg gag cat cgg aca
Mut2	gat cgg gtg tgg gtg gcg taa agg gag cat cgg
Mut3	gat cgg gtg tgg gtg gcg taa agg gag ca
Mut4	gat cgg gtg tgg gtg gcg taa agg gag c
Mut5	gat cgg gtg tgg gtg gcg taa agg gag

Fluorescent anisotropy measurement demonstrated (Fig. s1) that deletion of nucleotides from 5'-tail (Mut1) and 3'-tail (Mut2 – Mut5) result to increase of dissociation constant of aptamer-OTA interaction. It's consistent with previously published data, see Table s2.

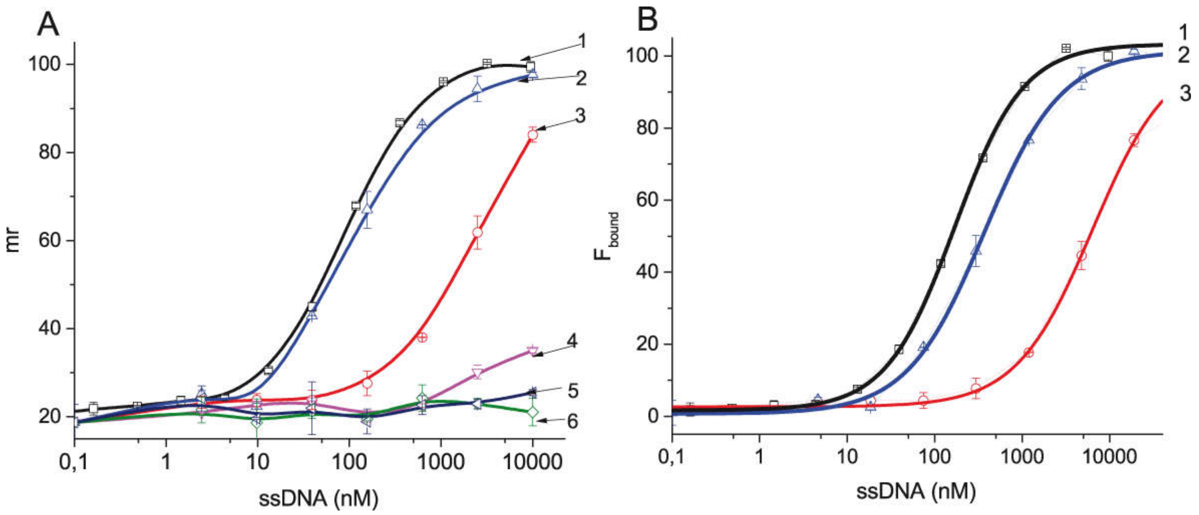

Figure S1 (A) Dependences of fluorescence anisotropy (mr) and (B) F_{bound} of OTA-Flu from aptamer and its shortened variants concentration, where1 – aptamer, 2 – Mut2; 3 – Mut1; 4 – Mut3, 5 – Mut4; 5 – Mut5

Table S2. Analysis of influence of mononucleotide mutation and deletion in aptamer 1.12.2 sequence on its ligand binding properties

Region	№ from 5' to 3'	Sequence	K_D (OTA-Flu), nM	K_D (OTA)**, nM	K_D (OTA)***, nM	K_D (OTA)***, nM
5'-tail	1	G	3300		2070	G to C – 900 / G to A - 1160 / G to T - 1470
	2	A			27220	
	3	T			NB	
	4	C				
	5	G				
G-core guanin	6	*G*				
	7	*G*				
Core loop	8	T				
	9	G				
	10	T				
G-core guanin	11	G				
	12	G				
Core loop	13	G				
	14	T				
G-core guanin	15	G				
	16	G				
Core loop	17	C				
	18	G				
	19	T				
	20	A				
	21	A				
	22	A				
G-core guanin	23	G				G to C - NB*
	24	G				G to C - 30000
3'-tail	25	G				
	26	A		NB*		
	27	G				
	28	C	NB*			
	29	A	NB*	NB*		
	30	T			20920	
	31	C	less than 10^{-6}	1600	423	
	32	G		500	148	
	33	G		400		
	34	A				
	35	C	192	200	46	
	36	A				
K_D with aptamer 1.12.2			183 nM	200 nM	55 nM	55 nM

*NB - no binding

** Data from [16]

*** Data from [19]

S2 Characterization of the interaction of the aptamer with complementary ssDNAs by isothermal microcalorimetry

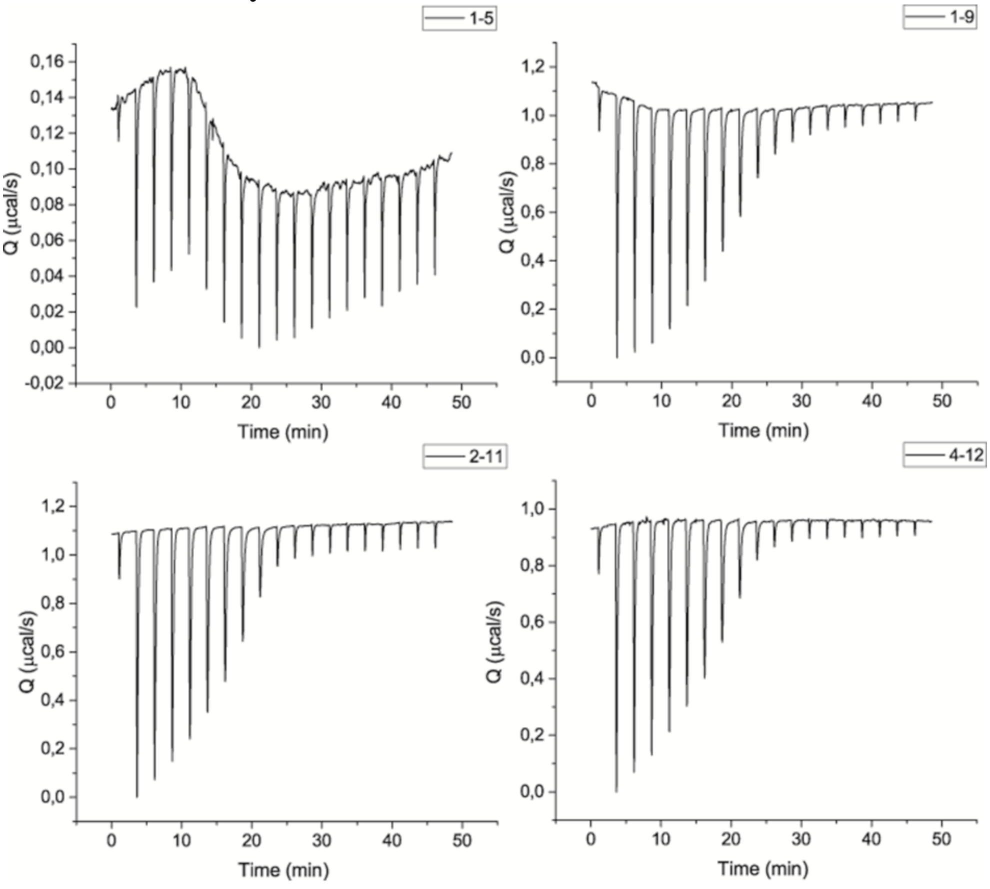

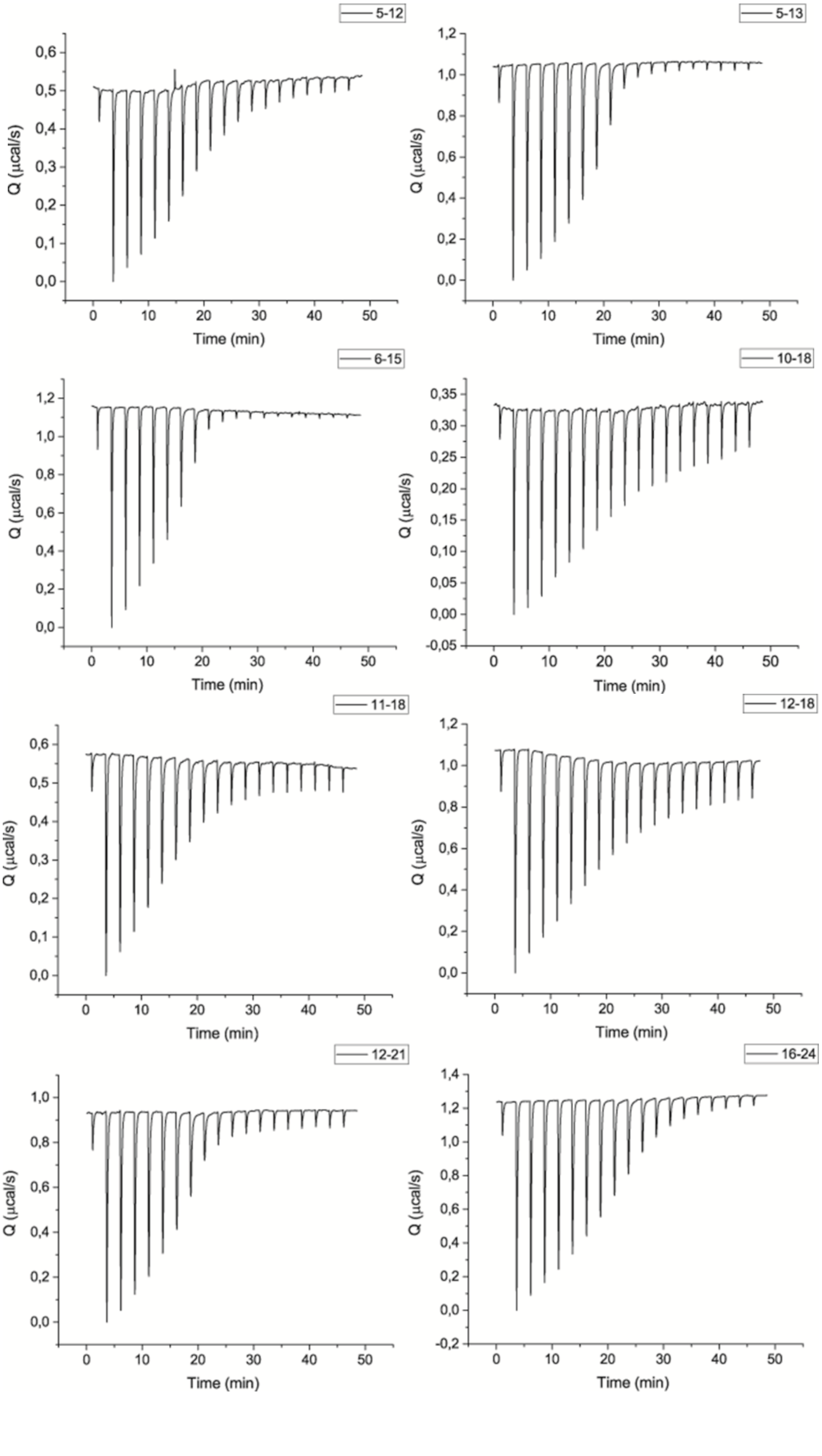
5-12
5-13
6-15
10-18
11-18
12-18
12-21
16-24
Q (μcal/s)
Time (min)

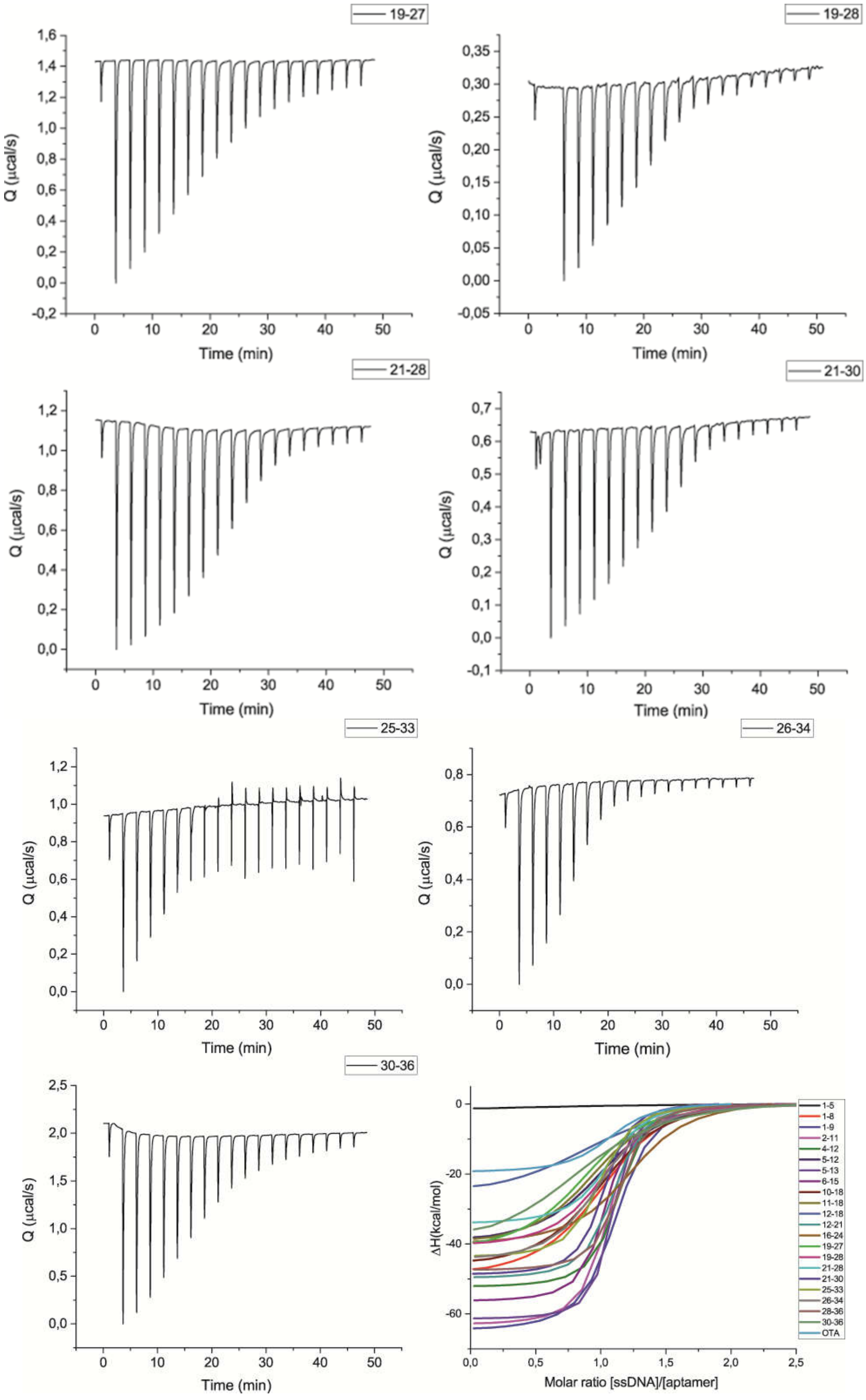

Figure S2. ITC measurements for sequential additions of ssDNA to aptamer and the integrated heat plots for all ssDNAs and for OTA.

S3 Characterization of ssDNAs interaction with aptamer in the presence of labeled OTA by fluorescence anisotropy

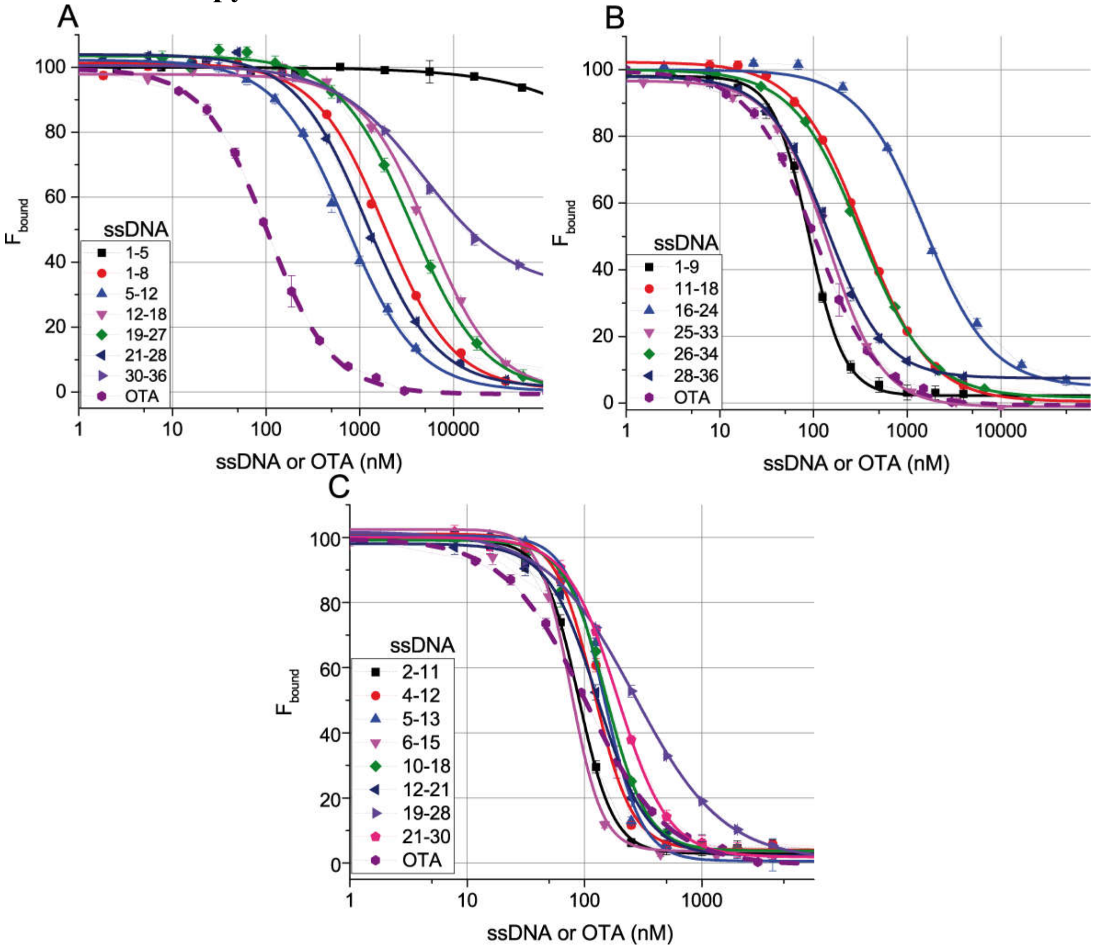

Figure S3 Dependences of the percentage of bound OTA-Flu from the concentration of ssDNA formed (A) from 13 to 22, (B) from 23 to 24 and (C) from 25 to 27 hydrogen bonds. $n = 3$

Table S3. Additional set of ssDNAs using to clarify the minimum number of H-bonds needed to influence aptamer-OTA interaction

Binding site	Sequence from 5'to 3'	Purines	Pyrimidines	Length bases	H-bond	K_I (nM)	Time (min.)
{18–25}	ccc ttt ac	4	4	8	20	NB*	NB*
{24–28}	gct cc	4	1	5	14	NB*	NB*
{28–33}	ccg atg	4	2	6	16	NB*	NB*

NB* – no binding detected

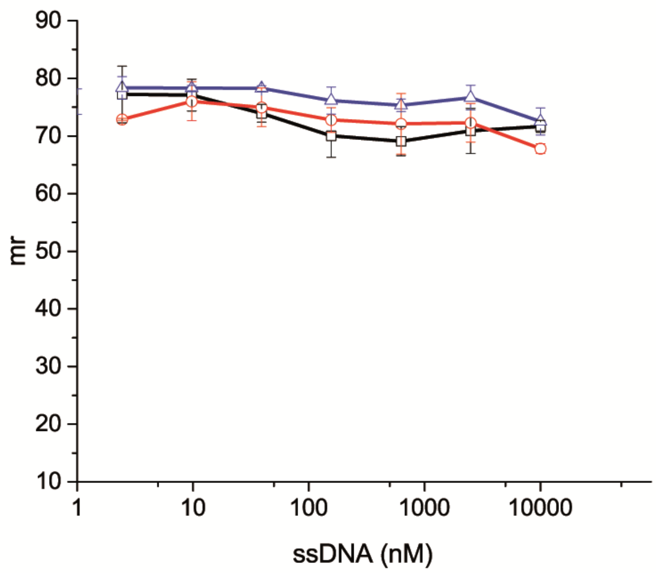

Figure S4 Dependences of the FA of OTA-Flu from the concentration of ssDNA {24–28} (square), {28–33} (circle) and {18–25} (triangle). n=3

S4 Characterization of the aptamer and its complexes with ssDNA by CD spectroscopy

Measurements of circular dichroism time dependences (Method)

Dependences of circular dichroism and absorbance from time were measured simultaneously at 290 nm for complexes aptamer–OTA, aptamer– {6–15}, and aptamer– {6–15} –OTA and at 260 nm for complexes aptamer– {28–36} and aptamer– {28–36} –OTA. Interval between the neighboring points was 5 sec, the measurement time varied from 360 sec to 4200 sec depends on the rate of equilibrium achievement during the complex formation. Measurements were carried out instantly after the injection of second (third, in case aptamer-OTA-ssDNA) component of the interaction without additional mixing. The data of all CD experiments were analyzed using the packaged Pro-Data Chirascan (Applied Photophysics, UK) and processed using the Origin 8.1 (Origin Lab, USA).

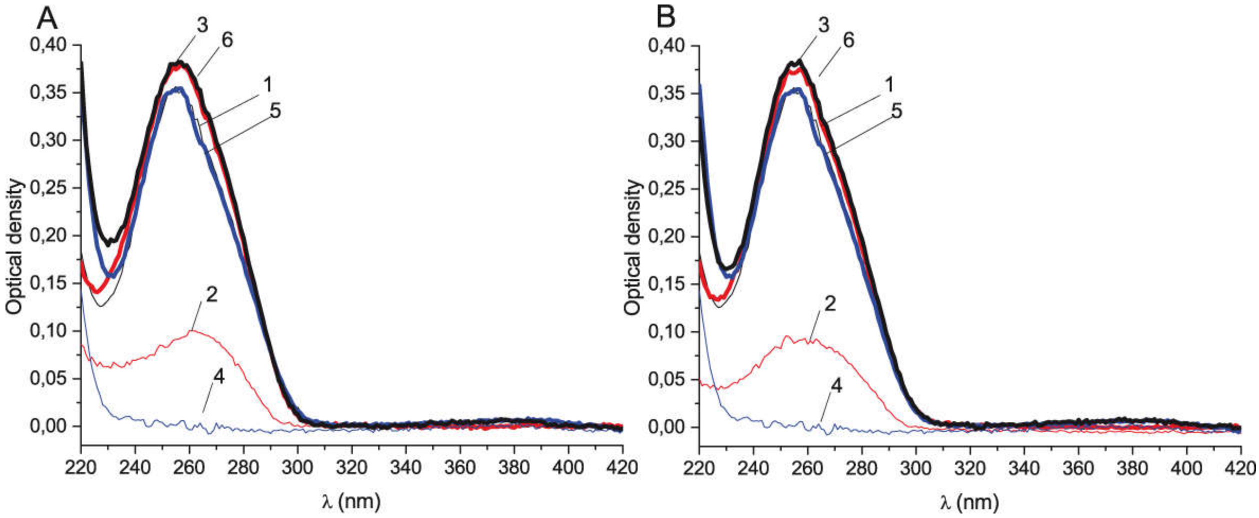

Figure S5 Absorbance spectra in equilibrium condition measured simultaneously with CD spectra (**A**) 1 – aptamer, 2 – ssDNA {6–15}, 3 – the aptamer–ssDNA {6–15}, 4 – OTA, 5 – the aptamer–OTA, and 6 – the aptamer–OTA complex after addition of ssDNA {6–15}. (**B**) 1 – aptamer, 2 – ssDNA {28–36}, 3 – the aptamer–ssDNA {28–36},4 – OTA, 5 – the aptamer–OTA, and 6 – the aptamer–OTA complex after addition of ssDNA {28–36}.

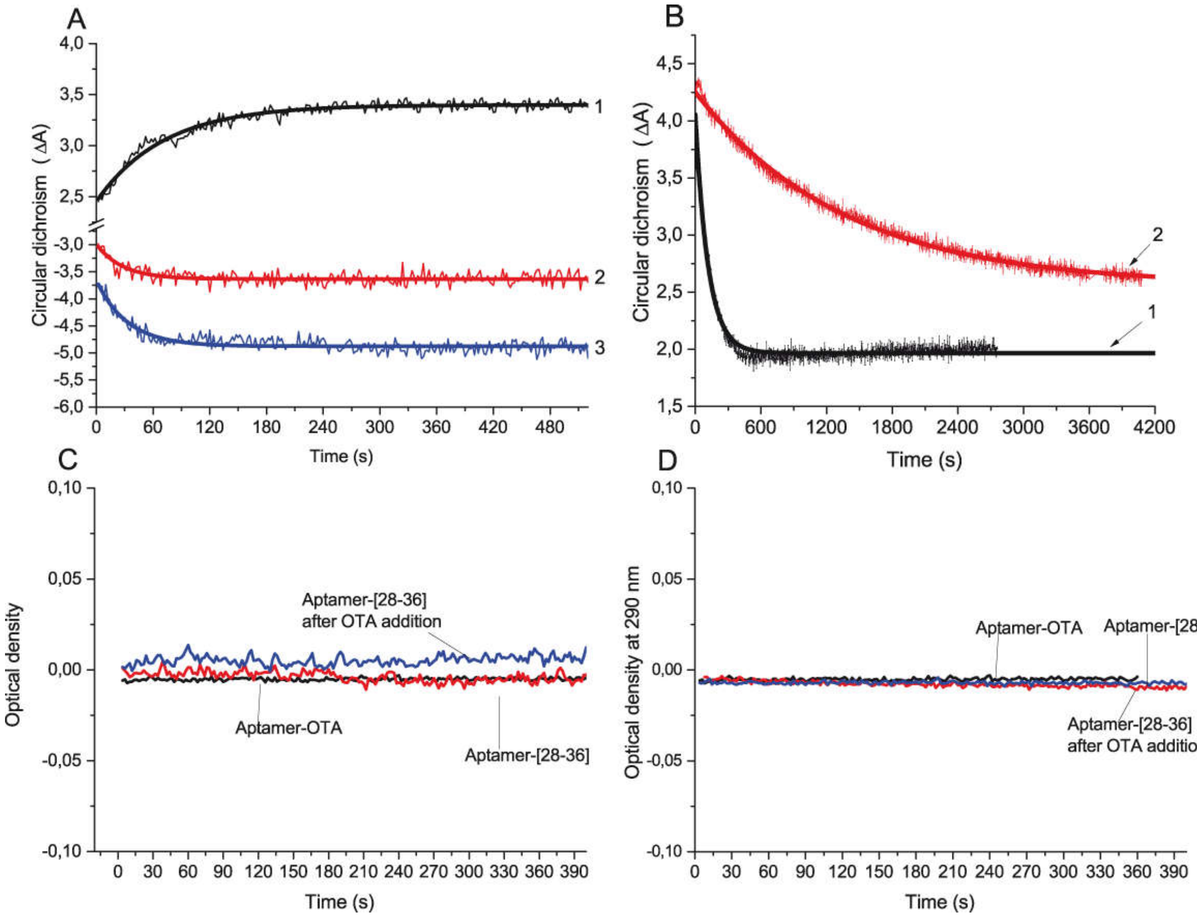

Figure S6 Time dependences of CD for the complexes: (A) 1 – aptamer–OTA at 290 nm, 2 – aptamer–{28–36} at 260 nm, and 3 – aptamer–{28–36} after addition of OTA at 260 nm; (B) 1 – aptamer– {5–16} at 290 nm and 2 – aptamer-OTA after addition of {5–16} at 290 nm. (C) Time dependences of absorbance for the complexes: aptamer–OTA at 290 nm, aptamer–{28–36} at 260 nm, and aptamer–{28–36} after addition of OTA at 260 nm (D) Time dependences of absorbance for the complexes: aptamer– {5–16} at 290 nm and aptamer-OTA after addition of {5–16} at 290 nm

Table S4. Additional CD extrema for the aptamer, OTA, ssDNAs and their complexes*.

Sample	Concentration (μM)	Extrema wavelength (nm)			
		«–»	«+»	«–»	«+»
Aptamer	1	– 236	246	– 261	292
OTA*	10	– 222	– 242	– 273	– 372
ssDNA {1–8}	2	– 239	274		
Aptamer + ssDNA {1–8}		243	276		
Aptamer + ssDNA {1–8} + 1 μM OTA		– 239	251	– 255	284
Aptamer + ssDNA {1–8} +10 μM OTA		– 233	251	– 260	288
ssDNA {1–9}	1	– 243	276		
Aptamer + ssDNA {1–9}		–245	269/278		
Aptamer + ssDNA {1–9} +1 μM OTA		– 245	281		
Aptamer + ssDNA {1–9} +10 μM OTA		– 231	251/–262		288
Aptamer + ssDNA {6–15} +10 μM OTA		– 240	266		
ssDNA {21–28}	1.5	– 232	277		
Aptamer + ssDNA {21–28}		– 238	255/–262		284
Aptamer + ssDNA {21–28}+1 μM OTA		– 233	248	– 261	287
Aptamer + ssDNA {21–28}+10 μM OTA		– 229	249	– 263	290
ssDNA {21–30}	1	– 248	276		
Aptamer + ssDNA {21–30}		– 240	284		
Aptamer + ssDNA {21–30}+1 μM OTA		– 238	248	– 254	288
Aptamer + ssDNA {21–30}+10 μM OTA		– 232	251	– 259	289

* for OTA all extrema are negative

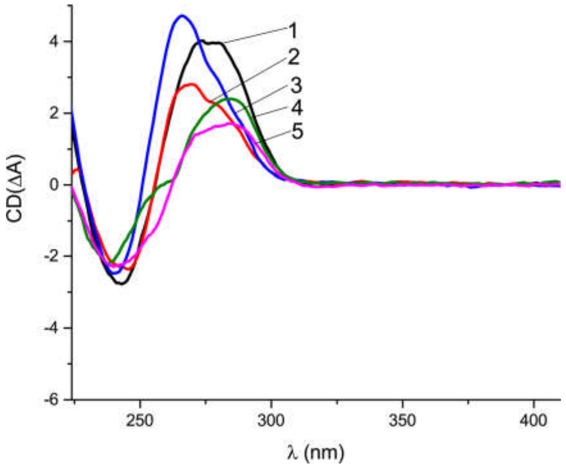

Figure S7 CD spectra of complexes of 1 μM aptamer with (1) 2 μM ssDNA{1–8}, (2) 1 μM ssDNA {1–9}, (3) 1 μM ssDNA {6–15}, (4) 1.5 μM ssDNA {21–28}, and (5) 1 μM ssDNA {21–30}.
